# SARS-CoV-2 Vaccines during Pregnancy and Breastfeeding: A Systematic Review of Maternal and Neonatal Outcomes

**DOI:** 10.3390/v14030539

**Published:** 2022-03-05

**Authors:** Domenico Umberto De Rose, Guglielmo Salvatori, Andrea Dotta, Cinzia Auriti

**Affiliations:** Neonatal Intensive Care Unit, Medical and Surgical Department of Fetus-Newborn-Infant, “Bambino Gesù” Children’s Hospital IRCCS, 00165 Rome, Italy; domenico.derose@opbg.net (D.U.D.R.); guglielmo.salvatori@opbg.net (G.S.); andrea.dotta@opbg.net (A.D.)

**Keywords:** neonates, COVID-19, infants, mothers, pregnancy, fetuses, miscarriage, malformations, women

## Abstract

**(1) Objective:** This systematic review summarizes current knowledges about maternal and neonatal outcomes following COVID-19 vaccination during pregnancy and breastfeeding. (**2) Study design:** PubMed, Cochrane Library, and the Education Resources Information Center (ERIC) were searched up to 27 October 2021. The primary outcome was to estimate how many pregnant and lactating women were reported to be vaccinated and had available maternal and neonatal outcomes. **(3) Results:** Forty-five studies sourcing data of 74,908 pregnant women and 5098 lactating women who received COVID-19 vaccination were considered as eligible. No major side-effects were reported, especially during the second and third trimester of pregnancy and during breastfeeding. Conversely, available studies revealed that infants received specific SARS-CoV-2 antibodies after maternal vaccination. **(4) Conclusions:** Vaccination against the SARS-CoV-2 virus should be recommended for pregnant women, after the pros and cons have been adequately explained. In particular, given the still limited evidence and considering that fever during the first months of gestation increases the possibility of congenital anomalies, they should be carefully counseled. The same considerations apply to breastfeeding women, also considering the immune responses that mRNA vaccines can generate in their human milk.

## 1. Introduction

Physiological, mechanical, and immunologic changes in pregnant women could influence their possibility of being attacked by the severe acute respiratory syndrome coronavirus 2 (SARS-CoV-2) [1]. Main symptoms of the disease (COVID-19) are related to an impaired microcirculatory function; indeed, infected pregnant women are at increased risk of preeclampsia (PE)-like symptoms [2] and of need for hospitalization and intensive care unit admission [3]. Given that the incidence of obstetrical complications, such as preterm birth, appears to be proportional to the severity of the infection, infants born to infected mothers with a more severe clinical course may have a worse outcome, mainly due to neonatal morbidity and mortality associated with prematurity. Therefore, the immunization of pregnant women against SARS-CoV-2 appears to be justified [4].

Although no conclusive evidence is yet completely available regarding the effectiveness and safety of COVID-19 vaccines in pregnancy, due to the non-inclusion of these women in clinical trials evaluating vaccines, the studies performed to date have allowed detecting a significant lower risk of contracting SARS-CoV-2 infection among vaccinated than unvaccinated pregnant women. The number of pregnant women vaccinated against COVID-19 to date, globally, has exceeded hundreds of thousands with no adverse event reports in excess of the nonpregnant population [5]. The same goes for the efficacy of vaccination during breastfeeding, which is considered to be similar to that among nonpregnant women. Currently there is unanimous consensus that there is no biological plausibility in support of a possible harm to infants nursed by vaccinated mothers [6]. Regarding the fertility of women who undergo vaccination against COVID-19, public health agencies and scientific societies internationally ruled out a possible association between vaccine and fertility problems [7].

**Figure 1:**
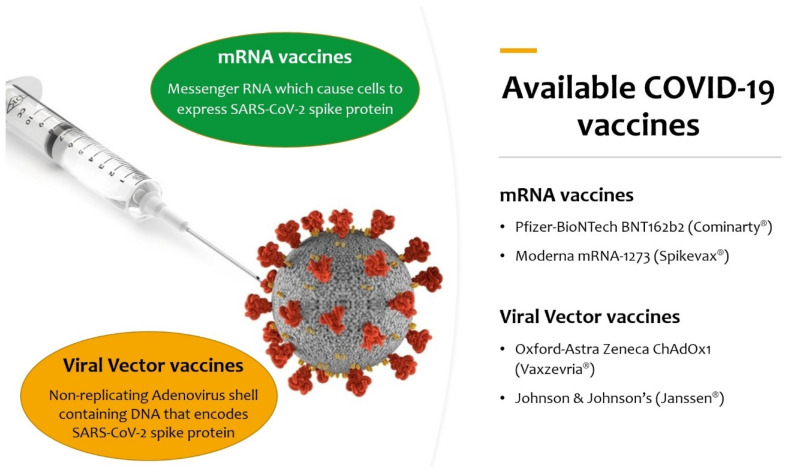
Available COVID-19 vaccines authorized for use by European Medicines Agency (EMA) at the moment of the literature search.

Our aim was to summarize the evidence in the literature on the outcomes of vaccination against SARS-CoV-2 in pregnant women and in women who have recently delivered, as well as the possible effects of vaccines currently used (Figure 1) on their newborns.

## 2. Materials and Methods

### 2.1. Search Strategy and Study Selection

This systematic review was performed following PRISMA guidelines [8]. Search terms included “SARS-CoV-2” OR “COVID-19” AND “vaccination” OR “vaccine” AND “pregnancy” OR “pregnant” OR “breastfeeding”. We considered studies providing information about maternal and/or child outcomes after maternal SARS-CoV-2 vaccination with a mRNA-based vaccine published after 1 January 2021. We did not apply limitations on study design to include all available literature, but preprint studies were not considered. The selection of studies was made through PubMed (http://www.ncbi.nlm.nih.gov/pubmed/, accessed on 27 October 2021), the Cochrane Library (https://www.cochranelibrary.com/advanced-search, accessed on 27 October 2021), and the Education Resources Information Center (ERIC, https://eric.ed.gov/, accessed on 27 October 2021) (Box 1).

The search was conducted as follows: Dr. De Rose (D.U.D.R.) identified relevant studies by reading the abstract and searching for additional studies through the reference lists of the selected papers. Then, Dr. De Rose (D.U.D.R.) and Dr. Auriti (C.A.) independently reviewed the studies by checking titles and abstracts of the articles and by deciding whether to include each article or not.

Box 1 **Different COVID-19 vaccine types to date.**

Different types of vaccines are under research to develop an effective vaccine against COVID-19:

- **mRNA vaccines:** based on messenger RNA (mRNA) or a self-replicating RNA that provides the genetic information required to produce the spike protein: Pfizer–BioNTech BNT162b2 (Comirnaty^®^) and Moderna mRNA-1273 (Spikevax^®^);
- **Viral-vector vaccines:** an existing virus that is incompetent for replication but contains DNA that encodes for the spike protein. In the case of ChAdOx-1S, developed by the University of Oxford and AstraZeneca (Vaxzevria^®^), the vector is a modified chimpanzee adenovirus; in the case of Johnson & Johnson’s Ad26.COV2.S (Janssen^®^), the vector is a recombinant human adenovirus (Ad 26–serotype 26); in the case of the Russian Gam-COVID-Vac (Sputnik V^®^), two recombinant replication-defective human adenoviruses were used (Ad26 and Ad5–serotype 5); in the case of Ad5-nCov (Convidecia^®^), the vector is similarly the human adenovirus serotype 5 (Ad5);
- **Recombinant protein vaccines:** based on the laboratory synthesis of the spike protein, the receptor-binding domain (RBD), or virus-like particles. This category includes the American Nuvaxovid^®^/Indian Covovax ^®^, the Russian EpiVacCorona^®^, the Chinese ZF2001 (Zifivax^®^), the Cuban Soberana-2^®^, and the Sanofi-GSK VAT00008;
- **Inactivated viral vaccines:** the SARS-CoV-2 virus has been cultivated in cell cultures and chemically inactivated. This category includes the Chinese CoronaVac^®^ and the Indian Covaxin^®^;
- **Live-attenuated virus vaccines:** a genetically weakened variant of the virus that replicates to a limited amount but does not cause illness while eliciting an immune response, as for measles, mumps, and rubella (MMR) vaccine;
- **DNA vaccines:** modified plasmids that carry genes that typically code for the spike protein, which is then produced in the vaccinated individual, as in Indian ZyCoV-D^®^ COVID vaccine.

**How do current available vaccines work?**

The Pfizer–BioNTech BNT162b2 vaccine and Moderna mRNA-1273 vaccine contain mRNA encoding for spike membrane proteins, encapsulated in lipid nanoparticles. Oxford–AstraZeneca ChAdOx1 contains SARS-CoV-2 virus cDNA fragments encapsulated in a viral vector. Both are non-replicants and self-destroying. When administered, macrophages and dendritic cells grab particles and convert them into proteins which are degraded into peptides and then exposed as antigens. Antigenic viral peptides are presented to major histocompatibility complexes (MHC) I and II and identified by helper T lymphocytes, causing B cells to synthesize neutralizing antibodies and cytotoxic T lymphocytes that kill infected cells.

Neutralizing antibodies directed to the virus membrane glycoproteins such as the spike protein and nucleocapsid proteins drive the humoral immune response to the SARS-CoV-2 virus. These antibodies prevent the virus from entering cells and, hence, its infectious capacity. However, not all antibodies have neutralizing activity, and some can increase the activity of the virus. Therefore, to solve this problem, mRNA fractions were isolated and encapsulated within lipid nanoparticles to create mRNA vaccines. COVID-19 infection is the first infection to be prevented with mRNA vaccines, and the vaccine underwent many tests quickly before being administered to humans. Both viral-vector vectors and mRNA vaccines induce a cell-mediated response by cytotoxic Th1 and CD8^+^ lymphocytes that recognize and digest viral antigens exposed by MCH class I molecules. As of yet, the induction of regulatory T cells in the vaccine-induced immune response has not been reported. Other COVID-19 vaccine types are now being studied in phase 1, 2, and 3 clinical trials (https://covid19.trackvaccines.org/vaccines/).

### 2.2. Assessment of Risk of Bias

We evaluated the quality of included cohort studies and the risk of bias using the Newcastle–Ottawa Scale (NOS). The NOS comprises “participant selection”, “comparability of study groups,” and “assessment of outcome or exposure”. A score above 6–7 denotes a reasonable quality.

## 3. Results

### 3.1. Study Selection Process

**Figure 2:**
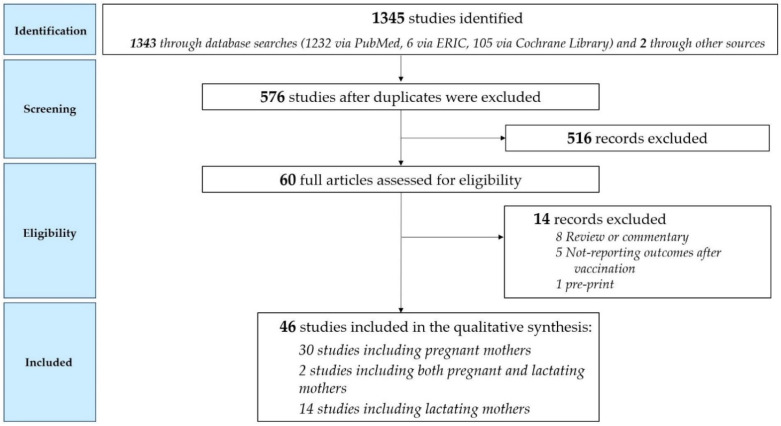
Study selection process.

The searches identified 1345 potentially relevant papers and studies, while 576 after duplicates were removed. After title and abstract screening, 60 full-text studies were considered potentially eligible for inclusion. A flowchart of study selection process is reported in Figure 2. We considered 46 records: 30 of them included pregnant mothers [9,10,11,12,13,14,15,16,17,18,19,20,21,22,23,24,25,26,27,28,29,30,31,32,33,34,35,36,37,38], two studies included both pregnant and lactating mothers [39,40], and 14 studies included lactating mothers [41,42,43,44,45,46,47,48,49,50,51,52,53,54]. Most studies were conducted in the United States (41.3%) and Israel (26.1%), along with three in Italy (6.5%), two in Spain (4.3%) and in Poland (4.3%), and the remaining (17.2%) in the United Kingdom, Qatar, Belgium, Germany, Norway, Portugal, the Netherlands, and Singapore.

**Figure 3:**
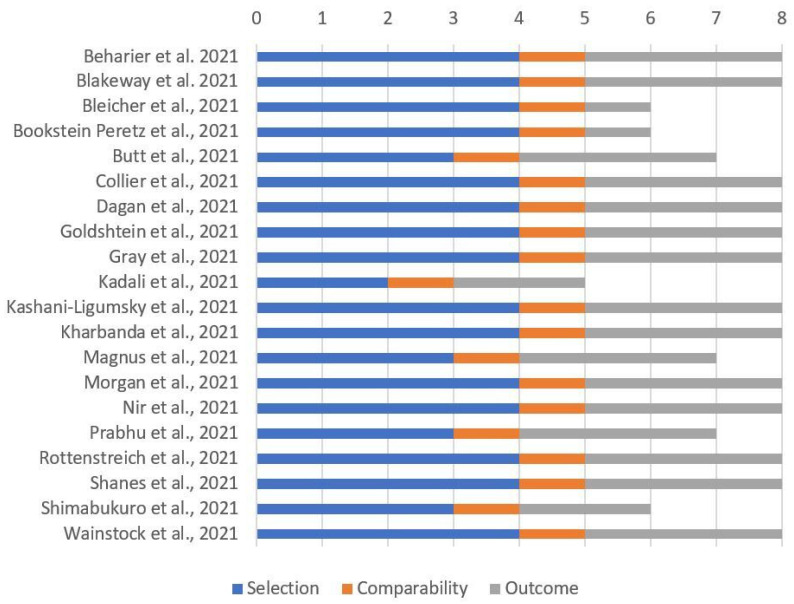
Quality assessment of included cohort studies involving pregnant women through “Newcastle–Ottawa Scale for cohort studies”.

Most cohort studies including pregnant women had a reasonable quality, as reported in Figure 3 (case series and case reports without a nonexposed group could not be considered).

**Figure 4:**
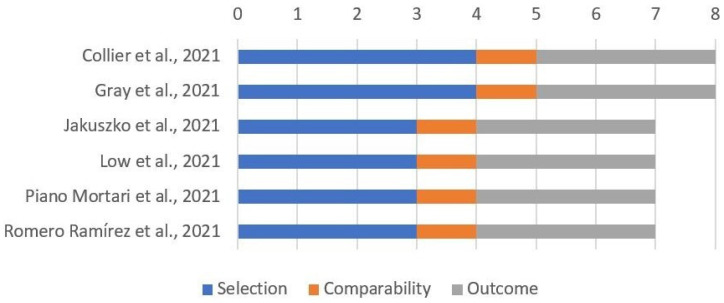
Quality assessment of included cohort studies involving breastfeeding mothers through “Newcastle–Ottawa Scale for cohort studies”.

Most studies including breastfeeding mothers reported only data about exposed women, without a control group; the remaining six studies had a reasonable quality, as reported in Figure 4.

### 3.2. Synthesis of Results in Pregnant Women

The characteristics and most relevant findings of the included studies about vaccination during pregnancy are reported in Table 1. The most relevant question concerns the safety of vaccination administered during pregnancy. No major adverse effects during pregnancy were reported.

To date, the largest study on the safety profile of mRNA vaccines during pregnancy is a cross-sectional survey published by Shimabukuro et al. in the New England Journal of Medicine, who reported preliminary findings from three US vaccine safety monitoring systems [34]. From 14 March 2020 to 28 February 2021 the authors surveyed over 35,000 women aged 16 to 54, identified as pregnant from their inclusion in various pregnancy registries. Among those women, 4000 provided information on the outcome; 827 of these delivered, and 29,000 reported post-vaccination symptoms. The limitation of the study is that all pregnant women were vaccinated in the third trimester, and this ruled out the possibility of evaluating some of the possible adverse effects of vaccinations on the progress of pregnancy and on newborns. However, although not fully comparable, the estimate of obstetrical and neonatal complications of maternal vaccination (*n* = 827) is similar to that described in studies on pregnant women conducted in the pre-COVID-19 period [30].

Comparing adverse effects in pregnant women and nonpregnant women, according Bookstein Peretz’s data, there were no additional adverse effects of vaccination during pregnancy. Furthermore, the rate and the severity of adverse effects were unaffected by the timing of immunization during pregnancy [12].

Goldshtein et al. confirmed this trend, with only 68/7530 women vaccinated during pregnancy (0.9%) reporting possible vaccine-related adverse events; none of them were serious. Headache (0.1%), overall asthenia (0.1%), unspecified pain (<0.1%), and stomachache (<0.1%) were the most often reported side-effects. Furthermore, mRNA immunization was linked to a considerably decreased probability of SARS-CoV-2 infection when compared to no immunization [19].

Two very large Israeli cohort studies provided data that support the safety of vaccination in pregnancy. The first is a retrospective study by Goldshtein et al. that looked at the relationship between immunization with Pfizer vaccine and the risk of infection in pregnant women who were vaccinated in the second and third trimesters of gestation. The primary outcome was SARS-CoV-2 infection demonstrated by the positivity of the molecular swab at 28 days after the initial vaccination dose. The cumulative incidence of infection in vaccinated women was significantly lower than in unvaccinated women. SARS-CoV-2 hospitalization rates were 0.2% in the protected women and 0.3% in the unprotected peers. During the study’s follow-up period, 18% of the vaccinated group and 18.9% of the unvaccinated group completed the pregnancy [19].

Dagan et al. in the second Israeli study compared a cohort of 10,861 pregnant women vaccinated with BNT162b2 mRNA matched 1:1 with 10,861 unvaccinated pregnant women. Twenty-six percent were immunized during the first trimester, 48% were immunized during the second trimester, and 26% were immunized during the third trimester. The results reflect the effectiveness of the vaccine against the alpha variant, which was predominantly circulating in Israel during the study period. The primary outcome was to determine the incidence of SARS-CoV-2 infection documented by a positive molecular swab and the incidence of symptoms and hospitalization. The periods in which the cumulative incidence was calculated were 14–20 days and 21–27 days following the first administration and 7–56 days following the second injection. The incidence of infection, as well as the risk of severe illness and hospitalization, was considerably greater in the unvaccinated group compared to the vaccinated group [15].

A very relevant question relates to the protective capacity of vaccination or natural infection for newborns. Several studies on this question have been performed. The most interesting was carried out in Boston on a cohort of 103 women protected with mRNA-1273 (Moderna) or BNT162b2 (Pfizer–BioNTech) vaccine, as well as 28 women with confirmed SARS-CoV-2 infection [39]: 30 vaccinated women were pregnant; 16 vaccinated women were post breastfeeding; 57 vaccinated women were neither pregnant nor breastfeeding. Among the 28 unvaccinated women, 22 were infected with SARS-CoV-2, while six were infected with SARS-CoV-2 and were breastfeeding. The authors measured the anti-SARS-CoV-2 receptor-binding domain (RBD) antibody titer and the neutralizing and non-neutralizing antibody titer after vaccination and after the natural infection. The presence of neutralizing and non-neutralizing antibodies and the response of CD4^+^ and CD8^+^ T-cells were detectable in all subgroups and were also observed in the umbilical cord blood and human milk following immunization. The level of antibodies against variants B.1.1.7 and B.1.351 was decreased, but the cellular T response was conserved against these variants.

The presence of neutralizing antibodies in pregnant women and newborns following vaccination was confirmed in all cohorts. Interestingly, a surprising discovery is that the transfer ratio appears to rise with latency from immunization; these findings imply that earlier immunization, at least among women in their third trimester, may create a stronger protection in the baby, the immunobiology of which deserves additional study [26].

### 3.3. Synthesis of Results in Lactating Women

Questions are often asked about the presence of antibodies and mRNA in breast milk after COVID vaccine. The characteristics and most relevant findings of the included studies on vaccination during breastfeeding are reported in Table 2. No major adverse reactions needing emergency treatment or hospitalization were described in mothers and infants.

It seems clear that, in the human milk of women protected with mRNA vaccines (currently preferentially indicated in Italy for women of reproductive age), there is a constant presence of anti-spike antibodies, particularly of the IgA and IgG type, as also shown by preliminary data from a study still in progress at the “Bambino Gesù” Children’s Hospital in Rome [53].

The relationship between milk IgA antibodies and immunization timing during breastfeeding has to be researched further, considering the drop in IgA levels recently observed of up to 25% in a cohort of lactating women (probably deriving from the different timing of withdrawals) [55].

To date, the largest study is a cross-sectional survey performed by McLaurin-Jiang et al., who recruited 4455 vaccinated breastfeeding mothers. According to their findings, vaccination appears to have few negative effects on lactation despite the reported short-term side-effects (such as fever, fatigue, or headache) [46], with resolution within 72 h after vaccination [51].

An increase in milk IgA levels is seen 2 weeks after the first dose of vaccine, and they are detectable in 86% of cases 2 weeks after the second dose; IgG was already detected just 1 week after the second dose [40,48,52].

When compared to detectable levels following the first dose, maternal blood IgG levels rose sixfold following the second dose; a similar pattern of increase was observed for milk IgG levels, although whether this could stimulate adequate infant immunity or not still requires further studies [55].

No mRNA presence was detected, highlighting the fact that there is no transfer of mRNA to the baby via breast milk and no reason to discontinue breastfeeding for this reason at the time of vaccine administration [44].

To exclude that mRNA vaccine components could get into milk after immunization, polyethylene glycol was measured by Golan et al. in milk samples collected before and after vaccine administration, and its concentration did not significantly change [55].

## 4. Discussion

Primary prevention of infections in pregnancy through vaccination is one of the most successful public health programs of the last 10 years. It has led to a significant decrease in maternal and perinatal morbidity and deaths from influenza and whooping cough [56]. The frequency of COVID-19 vaccination is still relatively low among pregnant women worldwide. Shamshirsaz et al. demonstrated that vaccination acceptance was significantly associated with the history of influenza or pertussis vaccine administration during pregnancy (OR = 3.03; 95% CI: 1.37–6.73; *p* = 0.006) [57].

Therefore COVID-19 infection in pregnancy has a course that is certainly more severe than in other periods of women’s life. It can cause adverse effects on the course of pregnancy, such as preterm birth, even if the real possibility and frequency of maternal–fetal transmission are still under study.

According to Ciapponi et al., COVID-19 pregnant women had a twofold higher risk of requiring mechanical ventilation, whereas their neonates had a threefold higher risk of being hospitalized in the neonatal intensive care unit (NICU) [58]. Kazemi et al. noted a marked risk of abortion in infected mothers; one possible mechanism could be placental inflammation induced by viral particles [59]. Most infections in neonates and children arise from family clusters [60], linked to infected adult patients; they often exhibit only milder clinical signs [61]. Recently, a few case reports of neonates presenting with a multisystem inflammatory syndrome after maternal SARS-CoV-2 infection (MIS-N) were reported [62].

Lumbreras-Marquez et al. assessed that, if all pregnant women received immunization during the two months of May and June 2021, the total expected maternal deaths in Mexico would have firmly decreased [63].

Data from mRNA vaccine studies show that the vaccine safety, tolerability, and efficacy in immunization are similar in pregnant women and their nonpregnant peers [4]. Since mRNA vaccines appear to stimulate Toll-like receptor 3 and that such activation has been associated with negative gestational outcomes [5], Shanes et al. examined the placentas of 84 women who got a SARS-CoV-2 vaccine during pregnancy and 116 unvaccinated women. The authors found no raised incidence of deciduous arterial disease, fetal vascular dysfunction, chronic low-grade villitis, or chronic histiocytic intervillositis in the vaccinated group [33].

However, the speed with which these vaccines were developed and authorized caused some concerns in the communities, starting from the possible effects on fertility or child outcomes. Long-term statistics are obviously scarce and will remain so for some years. The COVID-19 Vaccines International Pregnancy Exposure Registry (C-VIPER) will systemically estimate the risk of obstetric outcomes, neonatal outcomes, and infant outcomes in pregnant women exposed to a COVID-19 vaccine from 30 days before to the first day of their last menstrual period to end of pregnancy compared to a matched unexposed group. To date, the estimated study completion date is 31 December 2025 [64].

Although pregnant women were excluded from the first trials, at the time of writing, there is absolutely no evidence or theoretical reason to believe that any of the COVID-19 vaccinations could impair future fertility to date [7].

Concerns have also been raised about the chance of spontaneous abortion (pregnancy loss that occurs at fewer than 20 weeks of gestational age), a frequent event that affects 11 to 22 percent of identified pregnancies. Analyzing the data of the CVC v-safe COVID-19 pregnancy registry, the cumulative risk was within the expected risk range when data were compared to two historical cohorts that represent the “normal” lower and upper ranges of spontaneous abortion risk [37].

This systematic review is limited by the statistical heterogeneity in results of previous studies (ranging from single case reports to multicenter studies) and the relatively small sample size of pregnant women available in the literature, considering that more than 9.5 billion shots of COVID-19 vaccines have already been administered globally, at the time of writing.

However, we described the outcomes of a global cohort of 74,908 pregnant women and 5082 lactating women who received COVID-19 vaccination, and this can be considered an adequate sample to rule out any adverse effect in them and their infants.

Scientific societies agree in declaring these vaccines safe during pregnancy and breastfeeding.

The American College of Obstetricians and Gynecologists (ACOG) recommends that individuals over the age of 12, including pregnant and lactating women, receive a COVID-19 vaccine or a series of vaccines [65]. The European Board and College of Obstetrics and Gynecology (EBCOG) also said that immunization should be offered to all pregnant women, after they have been adequately informed of the benefits, especially in pregnant women at high risk for the presence of comorbidities (obesity, diabetes, heart disease, lung disease). Furthermore, the EBCOG recommends immunization against COVID-19 for all breastfeeding women, in the absence of a particular contraindication [6].

Considering the still limited evidence of vaccine safety during the first trimester of pregnancy, the Italian National Institute of Health and the Italian Obstetric Surveillance System recently updated guidelines for COVID-19 vaccinations during pregnancy; they cautiously stated that pregnant women who desire to be vaccinated should consider benefits and risks, given that fever is one of the possible reactions to the vaccine [66], and that this can cause an increased risk of birth defects [67], more so than the vaccine itself.

## 5. Conclusions

In this review we summarize the current knowledge about maternal and neonatal outcomes after COVID-19 vaccination, in order to help clinicians be thoroughly informed and fight misinformation. Benefits of COVID-19 vaccination outweigh the risks during pregnancy for both mothers and infants. Indeed, available studies report a good maternal immune response, as well as the transfer of maternal antibodies to confer passive protection against SARS-CoV-2 in newborns following maternal vaccination. The existence of anti-SARS-CoV-2 antibodies in breast milk suggests a possible specific protective effect on the newborn-infant after both maternal infection and vaccination, even if only clinical trials can provide scientific evidence.

It should be noted that the ability to protect oneself is not limited to the presence of circulating antibodies; next to these there are memory cells, which play a fundamental role in the response against SARS-CoV-2 in the event of virus exposure. Therefore, the potentiality of the cellular component (primarily lymphocytes) of breast milk to react to SARS-CoV-2 must be investigated.

## Figures and Tables

**Table 1:** List of the 32 studies including pregnant mothers who received SARS-CoV-2 vaccination.

First Author, Year	Country, Design	Type of Vaccine (Doses Given)	Vaccinated Pregnant Women (*n*)	First Vaccine Dose GA*	Outcome	Conclusions
Beharier, 2021 [9]	Israel, prospective, multicenter	Pfizer–BioNTech BNT162b2 (2 doses)	86	Mean ± SD: 34.5 ± 7.5	Antibodies in maternal blood and in umbilical cord blood	Strong maternal humoral IgG response (anti-S and RBD) that crosses the placenta barrier and approaches maternal titers in the fetus within 15 days following the first dose.
Blakeway, 2021 [10]	United Kingdom, retrospective, single-center	Pfizer–BioNTech BNT162b2 or Moderna mRNA-1273 or Oxford–AstraZeneca ChAdOx1 (at least one dose)	140	Second or third trimester	Uptake of COVID-19 vaccination and perinatal safety outcomes	Lower uptake among younger women, non-White ethnicity, and lower socioeconomic background. In a propensity score-matched cohort, the rates of adverse pregnancy outcomes were similar to that of unvaccinated pregnant women: stillbirth, fetal abnormalities, postpartum hemorrhage, cesarean delivery, small for gestational age, maternal high-dependency unit or ICU admission, or NICU admission.
Bleicher, 2021 [11]	Israel, an interim analysis of a prospective study	Pfizer–BioNTech BNT162b2 (at least one dose)	80	1st trimester (17.8%), 2nd trimester (54.5%), 3rd trimester (27.7%)	Complications in vaccinated and nonvaccinated pregnant women considered any of the following: vaginal bleeding, pregnancy loss, hypertension, gestational diabetes, and preterm birth	mRNA vaccine during pregnancy seems not to increase the rate of pregnancy complications and is effective in prevention of COVID-19 infection.
Bookstein Peretz, 2021 [12]	Israel, prospective, single-center	Pfizer–BioNTech BNT162b2 (2 doses)	57	Median 32.4 (IQR 31.2–33.6)	Vaccine-induced immunity and adverse events associated with the BNT162b2 vaccine among pregnant women	Favorable short-term obstetric and neonatal outcomes. The vaccine is effective in inducing humoral immunity in pregnant women, although SARS-CoV-2 IgG levels were lower when compared with those in nonpregnant vaccinated women. None of the pregnancies were complicated by fetal or neonatal death, and two (3.5%) neonates required NICU admission for respiratory support.
Butt, 2021 [13]	Qatar, retrospective, single-center	Pfizer–BioNTech BNT162b2 or Moderna mRNA-1273 (2 doses)	407	1st trimester (79.4%) or 2nd trimester (20.6%)	Vaccine effectiveness of mRNA vaccines in preventing confirmed SARS-CoV-2 infection during pregnancy >14 days after the second dose of the vaccine	Vaccine effectiveness was 86.8% (95% CI: 47.5–98.5) ≥14 days after the second dose. In the test-negative analysis, vaccine effectiveness >14 days after the first dose but before the second dose was 40.8% (95% CI: 0.0–80.4).
Cassaniti, 2021 [14]	Italy, retrospective, single-center	Pfizer–BioNTech BNT162b2 (2 doses)	2	31^+4^ and 27^+6^	Neutralizing antibodies in pregnant women and newborns	Antibody transfer occurred efficiently from mothers showing anti-SARS-CoV-2 IgG at delivery (elicited either by infection or by vaccination). However, the median neutralizing titer was twofold reduced in newborns with respect to mothers. This may be due to the contributions to neutralization in maternal serum of spike-specific IgA levels, which are not transmitted to the fetus.
Collier, 2021 [39]	Israel, prospective, single-center	Pfizer–BioNTech BNT162b2 or Moderna mRNA-1273 (2 doses)	30	1st trimester (17%), 2nd trimester (50%), 3rd trimester (33%)	Immunogenicity of the current COVID-19 mRNA vaccines in pregnant and lactating women against both the original SARS-CoV-2 USA-WA1/2020 strain and the B.1.1.7 and B.1.351 variants of concern	Binding, neutralizing, and functional non-neutralizing antibody responses, as well as CD4 and CD8 T-cell responses, were present in pregnant, lactating, and nonpregnant women following vaccination. Binding and neutralizing antibodies were also observed in infant cord blood and breast milk. Binding and neutralizing antibody titers against the SARS-CoV-2 B.1.1.7 and B.1.351 variants of concern were reduced, but T-cell responses were preserved against viral variants.
Dagan, 2021 [15]	Israel, prospective, single-center	Pfizer–BioNTech BNT162b2 (2 doses)	10,861	NA	Documented infection with SARS-CoV-2, symptomatic COVID-19, COVID-19-related hospitalization, severe illness, and death	High vaccine effectiveness of BNT162b2 was documented in pregnant women: estimated vaccine effectiveness from 7 through to 56 days after the second dose was 96% (95% CI: 89–100%) for any documented infection, 97% (91–100%) for infections with documented symptoms and 89% (43–100%) for COVID-19-related hospitalization. No deaths were observed.
Douxfils, 2021 [16]	Belgium, case report	Pfizer–BioNTech BNT162b2 (2 doses)	1	25	Neutralizing capacity of umbilical cord blood compared to maternal blood	Successful maternal to fetal transfer of neutralizing antibodies after vaccination with BNT162b2 in a pregnant woman at 25 weeks of gestation. The levels of neutralizing antibodies were approximately fivefold higher in the umbilical cord than in the maternal blood, while the level of total antibodies showed only a twofold increase.
Gill and Jones, 2021 [17]	United States, case report	Pfizer–BioNTech BNT162b2 (2 doses)	1	32^+6^	Antibodies in maternal blood and in umbilical cord blood	Vaccination in pregnancy produced a robust immune response for the patient, with subsequent transplacental transfer of neutralizing antibodies
Gloeckner, 2021 [18]	Germany, retrospective, single-center	Pfizer–BioNTech BNT162b2 or Moderna mRNA-1273 after a prime vaccination with Oxford–AstraZeneca ChAdOx1 (boost vaccination with a dose of mRNA vaccine after a prime vaccination with a vector-based vaccine)	3	NA	Antibody kinetics following heterologous vaccination in pregnant women in comparison to their newborns, as well as to a healthy nonpregnant control group	Vaccine induced SARS-CoV-2 spike IgG antibodies after vector-based prime vaccination in pregnancy, with an average increase of more than one log10 level after an mRNA-based boost. No significant differences were found compared with nonpregnant controls. They found similar levels of anti-spike IgG antibodies with a high neutralization capacity in the cord serum, indicating a strong passive humoral immunity in the newborns.
Goldshtein, 2021 [19]	Israel, retrospective, multi-center	Pfizer–BioNTech BNT162b2 (at least one dose)	7530	2nd and 3rd trimester	Documented SARS-CoV-2 infection 28 days or more after the first vaccine dose	BNT162b2 mRNA vaccination compared with no vaccination was associated with a significantly lower risk of SARS-CoV-2 infection. For 28 days or more postvaccination, a statistically significant hazard reduction was observed among the vaccinated group compared with the unvaccinated group (aHR = 0.22; 95%CI: 0.11–0.43; robust *p* < 0.001)
Gray, 2021 [40]	United States, retrospective, multi-center	Pfizer–BioNTech BNT162b2 or Moderna mRNA-1273 (2 doses)	84	23.2 (IQR 16.3–32.1)	Vaccine-induced immunity in vaccinated pregnant and lactating women	Robust and comparable IgG titers were observed across pregnant, lactating, and nonpregnant controls, all of which were significantly higher than those observed in pregnant women with previous SARS-CoV-2 infection. Boosting resulted in augmented IgG levels in the blood, translating to transfer of IgG to the neonate through the placenta and breast milk.
Kadali, 2021 [20]	United States, prospective, single-center	Pfizer–BioNTech BNT162b2 or Moderna mRNA-1273 (at least one dose)	38	NA	Side-effect profile of the mRNA vaccines among pregnant healthcare workers (HCWs) with that of nonpregnant HCWs	No significant statistical differences were found between the groups for all of the symptoms reported for both groups (however, the participant with a report of seizure had a known history of seizure disorder and borderline low anticonvulsant blood levels).
Kashani-Ligumsky, 2021 [21]	Israel, prospective, single-center	Pfizer–BioNTech BNT162b2 (2 doses)	29	3rd trimester	Titers of IgG antibodies to SARS-CoV-2 in umbilical cord blood in vaccinated pregnant women	Neonates born to mothers vaccinated during pregnancy had higher antibody titers and may, therefore, have more prolonged protection compared to those born to women infected during pregnancy.
Kharbanda, 2021 [22]	United States, retrospective case-control surveillance analysis of CDC Vaccine Safety Datalink	Pfizer–BioNTech BNT162b2 or Moderna mRNA-1273 or Janssen vaccine (at least one dose)	21,267	6–19	Case–control surveillance of COVID-19 vaccination during pregnancy and spontaneous abortion	Spontaneous abortions did not have an increased odds of exposure to a COVID-19 vaccination in the prior 28 days compared with ongoing pregnancies (adjusted odds ratio, 1.02; 95% CI: 0.96–1.08). Results were consistent for mRNA-1273 and BNT162b2 and by gestational age group.
Magnus, 2021 [23]	Norway, case-control, multi-center	Pfizer–BioNTech BNT162b2 or Moderna mRNA-1273 or Oxford–AstraZeneca ChAdOx1 (at least one dose)	772	NA	Previous COVID-19 vaccination and first-trimester miscarriage risk among women who had a miscarriage before 14 weeks of GA	No evidence of an increased risk for early pregnancy loss after COVID-19 vaccination.
Mangat, 2021 [24]	United States, case report	Pfizer–BioNTech BNT162b2 (2 doses)	1	22	Antibodies in neonatal blood	Persistence of anti-SARS-CoV-2 S antibodies was noted in a preterm infant at 6 months of age, which correlates with the prevention of COVID-19 and its complications in early infancy.
Mehaffey, 2021 [25]	United States, case report	Pfizer–BioNTech BNT162b2 (2 doses)	1	29	Antibodies in maternal and umbilical cord blood	Vertical transmission of IgG SARS-CoV-2 specific antibodies from a vaccinated mother to her son with no evidence of prior infection.
Mithal, 2021 [26]	United States, prospective, single-center	Pfizer–BioNTech BNT162b2 or Moderna mRNA-1273 (at least one dose)	27	33 ± 2	Transfer of SARS-CoV-2 IgG to infants following maternal vaccination during pregnancy	Most pregnant women who received a mRNA vaccine during the third trimester had transplacental transfer of IgG to the infant. The observed mean IgG transfer ratio demonstrated that infant antibody levels are about equal to the maternal levels.
Morgan, 2021 [27]	United Sates, retrospective, single-center	Pfizer–BioNTech BNT162b2 or Moderna mRNA-1273 or Janssen vaccine (at least one dose)	1332	NA	Incidence of severe or critical COVID-19 in vaccinated compared with unvaccinated pregnant patients in the context of Delta variant Predominance	Association between SARS-CoV-2 vaccination and lower odds of severe or critical COVID-19 and COVID-19 of any severity in pregnant patients during the Delta variant–predominant fourth surge of SARS-CoV-2.
Nir, 2021 [28]	Israel, prospective, single-center	Pfizer–BioNTech BNT162b2 (2 doses)	64	Mean ± SD: 33.5 ± 3.2 weeks at second dosage	Antibodies in maternal blood and in umbilical cord blood	SARS-CoV-2 IgG antibodies were detected in cord blood, newborn dried blood spot, and breast milk samples. Neonatal and breast milk antibody levels were positively correlated with maternal serum antibody levels. Higher levels of cord blood antibodies were detected in vaccinated women than in COVID-19-recovered women.
Paul, 2021 [29]	United States, case report	Moderna mRNA-1273 (one dose)	1	26	Antibodies in umbilical cord blood after maternal vaccination	SARS-CoV-2 IgG antibodies were detectable in a newborn’s cord blood sample after only a single dose of the Moderna COVID-19 vaccine.
Prabhu, 2021 [30]	United States, prospective, single-center	Pfizer–BioNTech BNT162b2 or Moderna mRNA-1273 (at least one dose)	122	NA	Antibodies in maternal blood and in umbilical cord blood	All women and cord blood samples, except for one, had detectable IgG antibodies by 4 weeks after vaccine dose 1. The increasing levels of maternal IgG over time and the increasing placental IgG transfer ratio over time suggest that timing between vaccination and birth may be an important factor to consider in vaccination strategies of pregnant women.
Rottenstreich, 2021 [31]	Israel, prospective, single-center	Pfizer–BioNTech BNT162b2 (at least one dose)	20	3^rd^ trimester	Antibodies in maternal blood and in umbilical cord blood	They demonstrated an efficient placental transfer of IgG antibodies following maternal SARS-CoV-2 vaccination, and a positive correlation between maternal and cord blood antibody concentrations. Nevertheless, while neonatal antibody levels were satisfactory, placental transfer ratios were relatively lower (0.44 for anti-S and 0.34 for anti-RBD IgG) as compared to prior studies of vaccine-elicited antibodies to influenza, pertussis, measles, rubella, and hepatitis B, in which transfer ratios ranging from 0.8 to 1.7 have been reported.
Rottenstreich, 2021 [32]	Israel, retrospective, multi-center	Pfizer–BioNTech BNT162b2 (at least one dose)	712	3rd trimester	Vaccination impact on adverse maternal and neonatal outcomes	The uptake of COVID-19 vaccination during the 3rd trimester of pregnancy was not associated with an increased risk of adverse maternal outcomes and lowered the risk for adverse neonatal outcomes
Shanes, 2021 [33]	United States, prospective, single-center	mRNA vaccine (not specified which, at least one dose)	84	NA	Frequency of placental lesions in patients who received SARS-CoV-2 vaccination in pregnancy	There was no observed increase in the incidence of findings characteristic of SARS-CoV-2 infection in pregnancy and no evidence of vaccine-triggered breakdown in maternal immunologic tolerance of the fetus.
Shimabukuro, 2021 [34]	United States, cross-sectional survey	Pfizer–BioNTech BNT162b2 or Moderna mRNA-1273 (at least one dose)	35,691 women	Periconception period (2.3%, 1st trimester (28.6%), 2nd trimester (43.3%), 3rd trimester (25.7%)	Participant-reported local and systemic reactogenicity to mRNA vaccines and pregnancy outcomes	Injection-site pain was reported more frequently among pregnant persons than among nonpregnant women, whereas headache, myalgia, chills, and fever were reported less frequently. Adverse neonatal outcomes included preterm birth (in 9.4%) and small size for gestational age (in 3.2%); no neonatal deaths were reported. Most abortions (92.3%) occurred before 13 weeks of gestation.
Trostle, 2021 [35]	United States, prospective, single-center	Pfizer–BioNTech BNT162b2 or Moderna mRNA-1273 (at least one dose)	36	1st trimester (6%), 2nd trimester (83%), 3rd trimester (11%)	Antibodies in maternal blood and in umbilical cord blood	Transplacental antibody transfer following mRNA COVID-19 vaccination during pregnancy, with 100% of cord blood specimens having high levels of anti-S antibodies.
Wainstock, 2021 [36]	Israel, retrospective, single-center	Pfizer–BioNTech BNT162b2 (at least one dose)	913	2nd or 3rd trimester	Associations among prenatal Pfizer–BioNTech COVID-19 vaccination, pregnancy Course, and outcomes	Prenatal maternal COVID-19 vaccine had no adverse effects on pregnancy course and outcomes.
Zauche, 2021 [37]	United States, retrospective analysis of CDC v-safe COVID-19 vaccine pregnancy registry	Pfizer–BioNTech BNT162b2 or Moderna mRNA-1273 (at least one dose either before conception or before 20 weeks of gestation)	2022	20	Cumulative risk of spontaneous abortion from 6 to less than 20 weeks of gestation	The cumulative risk of spontaneous abortion from 6 to less than 20 weeks of gestation was 14.1% (95% CI: 12.1–16.1) in the primary analysis and 12.8% (95% CI: 10.8–14.8) in an analysis using direct maternal age standardization to the reference population. As compared with data from two historical cohorts that represent the lower and upper ranges of spontaneous abortion risk, the risk of spontaneous abortion after mRNA COVID-19 vaccination either before conception or during pregnancy was consistent with the expected risk of spontaneous abortion; these findings add to the accumulating evidence about the safety of mRNA COVID-19 vaccination in pregnancy.
Zdanowski, 2021 [38]	Poland, retrospective, single-center	Pfizer–BioNTech BNT162b2 (2 doses)	16	31.8 ± 2.1	Antibodies in maternal blood and in umbilical cord blood	High titers of anti-S antibodies in cord blood after birth, suggesting that maternal immunization may provide protection to newborns through the transplacental transfer of antibodies. The trend in the correlation coefficients of the number of weeks from the first vaccine dose to delivery is worth noting.

* GA: gestational age.

**Table 2:** List of the 16 studies including pregnant mothers who received SARS-CoV-2 vaccination.

First Author, Year	Country, Design	Type of Vaccine (Doses Given)	Vaccinated Lactating Women (*n*)	Outcome	Conclusions
Baird, 2021 [41]	United States, prospective, single-center	Pfizer–BioNTech BNT162b2 or Moderna mRNA-1273 (2 doses)	7	Antibodies in human milk	Significantly elevated levels of SARS-CoV-2-specific IgG and IgA antibodies in human milk beginning approximately 7 days after the initial vaccine dose, with an IgG-dominant response.
Bertrand, 2021 [42]	United States, prospective, single-center	Pfizer–BioNTech BNT162b2 or Moderna mRNA-1273 (2 doses)	180	Safety of vaccination in breastfeeding women and their breastfed children	More than 85% of 180 breastfeeding women who received an mRNA COVID-19 vaccine reported local or systemic symptoms, with higher frequency following the second dose, but no serious adverse events were noted. Some women reported a temporary reduction in milk supply, but the milk supply returned to normal within 3 days. Moreover, few women reported an increase in milk supply after each dose.
Charepe, 2021 [43]	Portugal, prospective, single-center	Pfizer–BioNTech BNT162b2 (2 doses)	14	Serological profile of lactating women compared to nonlactating women, after immunization with the BNT162b2 Pfizer vaccine, in a cohort of healthcare workers, and antibody transfer via breast milk	All women showed immunity after vaccination with positive antibodies for IgM, IgA, and IgG antibodies. The dominant serum antibody response was IgG. Modest levels of antibodies in the breast milk of lactating mothers were observed in this study, especially IgG in 42.9%.
Collier, 2021 [39]	Israel, prospective, single-center	Pfizer–BioNTech BNT162b2 or Moderna mRNA-1273 (2 doses)	16	Immunogenicity of the current COVID-19 mRNA vaccines in pregnant and lactating women against both the original SARS-CoV-2 USA-WA1/2020 strain and the B.1.1.7 and B.1.351 variants of concern	Binding, neutralizing, and functional non-neutralizing antibody responses, as well as CD4 and CD8 T-cell responses, were present in pregnant, lactating, and nonpregnant women following vaccination. Binding and neutralizing antibodies were also observed in infant cord blood and breast milk. Binding and neutralizing antibody titers against the SARS-CoV-2 B.1.1.7 and B.1.351 variants of concern were reduced, but T-cell responses were preserved against viral variants.
Golan, 2021 [44]	United States, prospective, single-center	Pfizer–BioNTech BNT162b2 or Moderna mRNA-1273 (at least one dose)	7	Detection of vaccine-related mRNA in human milk after vaccination	Vaccine-associated mRNA was not detected in 13 milk samples collected 4 to 48 h after vaccination from 7 breastfeeding individuals.
Gray, 2021 [40]	United States, prospective, multi-center	Pfizer–BioNTech BNT162b2 or Moderna mRNA-1273 (2 doses)	31	Vaccine-induced immunity in vaccinated pregnant and lactating women	Robust and comparable IgG titers were observed across pregnant, lactating, and nonpregnant controls, all of which were significantly higher than those observed in pregnant women with previous SARS-CoV-2 infection. Boosting resulted in augmented IgG levels in the blood, translating to transfer of IgG to the neonate through the placenta and breast milk.
Guida, 2021 [45]	Italy, prospective, single-center	Pfizer–BioNTech BNT162b2 (2 doses)	10	Release of SARS-CoV-2 Spike (S) antibodies in human milk samples obtained by patients vaccinated with the mRNA BNT162b2 vaccine	Seven days after the 2nd dose, anti-SARS-CoV-2 S antibodies were detected in all sera and in all milk samples. The milk antibodies/serum antibodies ratio was on average 2%.
Jakuszko, 2021 [46]	Poland, prospective, single-center	Pfizer–BioNTech BNT162b2 (2 doses)	32	Immune response to vaccination against COVID-19 in breastfeeding women and possible benefits for both mother and child	As there were no serious side-effects in the children after the mothers’ vaccinations, and the presence of IgG and IgA antibodies in the breast milk was confirmed, the study gives further evidence on the importance of vaccination against COVID-19 in breastfeeding women.
Juncker, 2021 [47]	The Netherlands, prospective, single-center	Pfizer–BioNTech BNT162b2 (at least one dose)	26	Levels of specific IgA antibodies in human milk following the first and second dose of BNT162b2	In human milk, a biphasic response was observed, with SARS-CoV-2 specific IgA starting to increase between days 5 –7 after the first dose and declining after day 15, on average. After the second dose, an accelerated immune reaction was observed.
Kelly, 2021 [48]	United States, prospective, single-center	Pfizer–BioNTech BNT162b2 (2 doses)	5	Antibodies in human milk	They characterized longitudinal breast milk levels of anti-spike IgG/A following BNT162b2 vaccination, demonstrating sustained elevation of IgG/IgA levels. However, individual-level data suggest a possible gradual decline in anti-spike IgA in human milk over time after the second dose.
Lechosa-Muñiz, 2021 [49]	Spain, prospective, single-center	Pfizer–BioNTech BNT162b2, Moderna mRNA-1273, or Oxford–AstraZeneca ChAdOx1 (2 doses of mRNA vaccines or just 1 dose of vector-based vaccine)	110	Presence of IgG and IgA antibodies directed against SARS-CoV-2 protein S in blood from breastfeeding women and to detect the presence of IgA and IgG isotype antibodies directed against SARS-CoV-2 protein S in breast milk	The anti-SARS-CoV-2 vaccines used were well tolerated by mothers and breastfed infants. Breastfeeding must not be interrupted after vaccination. They showed a positive correlation between antibody levels in serum and breast milk samples (lower in who received AstraZeneca). As an added value, breastfeeding mothers offer their infants IgA and IgG isotype antibodies directed against SARS-CoV-2 protein S in breast milk.
Low, 2021 [50]	Singapore, prospective, single-center	Pfizer–BioNTech BNT162b2 (2 doses)	14	Production and secretion of spike- and receptor-binding domain (RBD)-specific IgA and IgG into human milk	Lactating mothers secreted specific IgA and IgG antibodies into milk, with the most significant increase at 3–7 days post dose 2. Virus-specific IgG titers were stable out to 4–6 weeks after dose 2. In contrast, SARS-CoV-2-specific IgA levels showed substantial decay. Infants who consumed post-vaccination human milk had no reported adverse effects up to 28 days post ingestion.
McLaurin-Jiang, 2021 [51]	United States, cross-sectional survey through social networks	Pfizer–BioNTech BNT162b2 or Moderna mRNA-1273 (at least one dose)	4455	Impact on breastfeeding of vaccine-related side-effects following COVID-19 vaccination	Minimal disruption of lactation or adverse impact on the breastfed child, more after the 2nd dose: 94% of women reported no changes in milk production or described an increase
Perl, 2021 [52]	Israel, prospective, single-center	Pfizer–BioNTech BNT162b2 (2 doses)	84	Antibodies in human milk and any potential adverse events among women and their infants	Specific IgA and IgG antibodies were found in human milk for 6 weeks after vaccination. IgA secretion was evident as early as 2 weeks after vaccination followed by a spike in IgG after 4 weeks (1 week after the second vaccine).
Piano Mortari, 2021 [53]	Italy, prospective, single-center	Pfizer–BioNTech BNT162b2 (2 doses)	16	Measurement of memory B cells (MBCs) and antibodies in human milk	Completing the vaccination cycle is necessary to generate high levels of specific serum antibodies and MBCs. Seven days after the second vaccine dose, all lactating mothers had detectable spike-specific IgA in human milk, confirming the ability of vaccine-induced MBCs to be home to the inflammatory environment of the lactating mammary gland and locally produce IgA.
Romero Ramírez, 2021 [54]	Spain, prospective, single-center	Pfizer–BioNTech BNT162b2 or Moderna mRNA-1273 (2 doses)	98	Antibodies in human milk	BNT162b2 and mRNA-1273 COVID-19 vaccines generate immunity in vaccinated mothers and are associated with vaccine-specific immunoglobulin concentrations in human milk.

## Data Availability

Not applicable.
